# Eye Injuries, with Special Reference to the Workmen's Compensation Act, 1906

**Published:** 1909-11-20

**Authors:** A. Maitland Ramsay

**Affiliations:** Ophthalmic Surgeon, Glasgow Royal Infirmary


### Hospital Clinics.

### EYE INJURIES, WITH SPECIAL REFERENCE TO THE WORKMEN'S COMPENSATION ACT, 1906.

By A. MAITLAND RAMSAY, M.D. ; Ophthalmic Surgeon, Glasgow Royal Infirmary.

(The Opening Address of the Paisley Medical Society, on November 11, 1909.)

(*Continued from page* 179.)

An accident involving a penetrating wound of the eye is always a matter of concern, because the doctor's ordinary sense of responsibility is intensified by the knowledge that the injury may be followed by sympathetic inflammation of the other eye, and that this may be so severe as ultimately to cause blindness. The medical expert has rarely anything to do with the treatment of the patient, but both in his report and in his evidence he is expected to give an opinion regarding the amount of damage to sight which is likely to result from the accident; and the prognosis in connection with penetrating wounds can, in most instances, be neither certain nor satisfactory, as long as there is danger of sympathetic disease.

Clinical experience justifies the division of cases of this affection into those in which there is sympathetic irritation and those in which there is sympathetic inflammation.

Sympathetic irritation is usually an early symptom, but it is often met with in cases where the injured eye has been blind for a long time and is undergoing degenerative changes. The patient feels that his eye gets soon tired; he has difficulty in reading small print; and after prolonged work he suffers from transitory attacks of dimness of vision when he looks at distant objects, or it may be from momentary total blindness. He feels uncomfortable in a bright light, which may cause neuralgic pains to dart through his head, and induce injection of the conjunctiva accompanied by copious lachrymation.

If the field of vision be at this stage carefully examined by Bjerrum's screen the blind spot in the sympathising eye will in many instances be found to have assumed a spindle-shape, and though I do not wish to insist too strongly on the value of this objective sign in the diagnosis of impending sympathetic mischief, I think it highly probable that, where there is an infected wound or degenerative changes in the other eye, it denotes active congestion of the optic disc, which may be regarded as a danger-signal indicating the approach of genuine sympathetic disturbance. At this stage, too, the patient is sometimes found to suffer from a low degree of myopia, and the co-existence of both signs greatly strengthens the diagnosis. This temporary myopia may be due to spasm, but it occasionally persists under atropine, and then it can be explained only by supposing that congestion of the choroid has brought about an altered state of the media, whereby the refractive index has been increased. Whatever be the true significance of these signs this much is certain, that both disappear after the removal of the exciting eye. For a time after enucleation the blind spot may retain the spindle form, and so long as this is the case the patient continues to complain that his vision is weak and uncertain, and that everything looked at appears "wavy" and "unsteady"; but all these symptoms pass away as soon as the blind spot assumes its normal shape. Associated with the indications just noted, ophthalmoscopic examination commonly reveals congestion of the optic disc and increased fulness and tortuosity of the retinal blood-vessels. It is, however, always a difficult matter, when there is no healthy fundus for comparison, to determine by the ophthalmoscope alone whether the optic disc is, or is not, congested, hence the value of the confirmatory evidence afforded by the co-existence of all these signs.

Sympathetic inflammation may or may not be preceded by the symptoms just described; usually failing sight is the first warning that the patient receives of the development of the disease. When the eye is examined a zone of pink hair-like vessels is seen surrounding the cornea, the iris is dull, and the pupil is small and sluggish, and dilates irregularly after the instillation of atropine. Even at this early stage there may be neuro-retinitis and floating bodies in the vitreous, but more frequently the details of the fundus are obscured by haziness of the media incidental to inflammation of the uveal tract. The cornea also becomes inflamed, spots form on its posterior surface—keratitis punctata—the aqueous is turbid, and the anterior chamber deep. The corneal signs are all the more marked when the inflammation assumes the serous type—serous iridocyclitis—but the plastic form is by far the more frequent and the more serious. Blood-vessels now develop upon the surface of the iris, whose substance thickens and bulges into the anterior chamber, the exudation filling up the pupil, and later on gluing the whole posterior surface of the iris to the capsule of the lens (complete posterior synechiæ), matting the ciliary processes together, and implicating the choroid so extensively that the nutrition of the eye is gravely affected. As a result, the iris is retracted at its ciliary attachment, the tension diminishes, the lens becomes cataractous, and the shrinking vitreous causes detachment of the retina and of the anterior portion of the choroid. Up to this time there has been perception of light, but now blindness becomes total. The eye is liable to recurrent attacks of iridocyclitis; its blood-vessels degenerate, and, rupturing, give rise to intra-ocular hæmorrhage; it steadily shrivels, and the final result is phthisis bulbi.

Since after sympathetic inflammation has fairly begun practically nothing can be done to check its progress or to repair the damage which it has caused, treatment, to be effective, must be prophylactic, and we know that in the stage of irritation, and even in the early stage of serous iridocyclitis in the previously healthy eye, the removal of the one that has been injured is followed by most beneficial results. Much of necessity depends on the manner in which the eye has been treated immediately after the accident, for we know that by skilful treatment eyes seriously injured may be saved and sympathetic inflammation prevented. In many cases enucleation is inevitable. It may happen, however, that a workman threatened with sympathetic mischief refuses to have the injured eye enucleated until it is too late to save sight. In these circumstances the employer may be placed in the position of having to make provision for life for an employee, whereas if the man had followed the surgeon's advice and submitted to enucleation he might have been able to resume his work as a one-eyed man. It is hard, legally, to compel anyone to part with an eye, but if, as a result of such a refusal to follow a surgeon's advice, sympathetic mischief ensue, then the workman, or, if he be a minor, his guardian, ought to sign a written declaration accepting full responsibility for the action.

When such a document is produced in court the Sheriff should have power not to penalise the employer for his servant's wilful disregard of competent advice. Under such circumstances the medical evidence would turn largely on what is authoritatively considered the recognised treatment of the case, and the expert will need to be very wary lest the counsel for the workman entrap him into saying anything which appears to cast discredit upon a fellow practitioner. He will also be expected to give his opinion whether the workman was acting reasonably or unreasonably in refusing operation, whether the operation was likely to be successful, and whether any, and if any then how much, risk to life or danger to health would attend it. In no case should a medical opinion be given unless the doctor has had an opportunity of making himself absolutely sure of the facts.

The question of operation may again arise if the workman suffer from traumatic cataract. The medical expert may be asked in court whether operation would be desirable, and whether the extraction of the cataract would restore the man to his former efficiency. The answer will depend on the amount of damage to the other tissues of the eye, as well as upon the occupation of the workman. If binocular vision be necessary for the satisfactory performance of his duties, surgical interference will be of little value to him, because, even although the operation were to be quite successful, full vision could only be obtained by wearing strong convex spectacles. Consequently single vision with both eyes open would no longer be possible. If, on the other hand, the workman depend on a wide visual field, extraction of the cataract will be of decided advantage to him. It will remove the blind side, and obviously to a coachman or to a carter this must be a great gain. It is well known that bottle-makers suffer from cataract more frequently than other persons, and when this affection was added to the list of industrial diseases for which compensation could be claimed it was explicitly stated that "the compensation should be made payable only in cases where an operation is undergone, and for a period not exceeding six months." A dispute may arise as to whether the cataract is really due to an injury or not, and the medical expert will be expected to settle the question. If the fact of an injury can be proved, and more especially if the patient be young and the cataract be confined to one eye, the probabilities are all in favour of its traumatic origin, and this will become a certainty if the iris of the affected eye be tremulous, or if there be a scar on the cornea, or any other sign of injury to the globe.

In every case in which a patient complains of failure or loss of sight, but in which there is no sign of injury to either the superficial or deep structures of the eyeball, a very guarded prognosis ought to be given. In the absence of any visible lesion the surgeon is wholly dependent upon the patient's testimony, which may be true or false. In such circumstances he ought to examine the eyes repeatedly in order that he may be either assured of the truth of the statements made to him or able to put an end, as promptly as possible, to any attempt at malingering. The framers of the Act doubtless foresaw that the liberal privileges which it conferred might tempt some to try to obtain the benefit for an undue length of time, therefore they inserted a clause making it incumbent on the workman to submit himself for examination by a duly qualified medical practitioner provided and paid by the employer. They protected the rights of the master still further by making it legal for him, in the event of the workman refusing to submit himself to such an examination, or putting obstructions in the way of its being made, to suspend payment of the weekly compensation until the medical examination has taken place. The workman may refuse to be examined by the doctor employed by the master if he think fit, but he cannot both object to be examined and at the same time enforce the weekly payments. When a medical expert is consulted in these circumstances he requires to take great care not to cast doubt on the veracity of a patient who is really suffering from defective sight. Cases of traumatic amblyopia undoubtedly occur, and it may be years after the accident before degenerative changes in the optic nerve can be detected. While, however, the surgeon should always be anxious to give the workman the benefit of the doubt, he must not shut his eyes to the fact that there are people who are unable to resist the temptation to make the most of an accident, and who, in striving to obtain greater pecuniary gain, make statements regarding their eyesight which they know to be false. To detect such deception is one of the most obvious and important duties of the surgeon. When the compensation provided by law is supplemented by sick allowance from one or more Friendly Societies, the workman may find himself so comfortable in idleness that he is in no hurry to resume his work. Prolonged idleness is good for no one, and in the case of the workman it is utterly demoralising. He becomes inclined to lounge and smoke all day, with the result that compensation is occasionally being paid when the defective sight is due not to the injury but to tobacco amblyopia. At times, in order to deceive the doctor, he applies irritants to the conjunctiva, or tries to make him believe that lesions of old standing are the direct result of a recent injury. It is in the interests not only of the employer but also of the employee that work be resumed as soon as possible. The performance of regular duties has always a distinct therapeutic value, and even although partial incapacity persist, it is better for the man to engage in some light occupation than to remain idle. If a man has lost an eye, or has had the sight of one eye seriously and permanently damaged, he, as a rule, refuses to return to work unless he receives a lump sum in lieu of the compensation to which he considers himself entitled. The Act, however, allows nothing in the nature of a solatium—an employer is not responsible for the results of an accident, except to the extent to which it produces physical incapacity. Much litigation would be avoided if a specified sum, varying in amount according to the workman's occupation, were to be fixed as the compensation to be paid in the event of the loss of an eye. A considerable proportion of the cases that now occupy the time of the Courts would then be settled without legal procedure. Even in ordinary circumstances the capacity for self-deception, especially in things medical, is great with most people, consequently it is not strange that the mental strain incidental to any medico-legal inquiry connected with the settlement of a claim for damages tends to make those who have no wish to deceive give an exaggerated account of their symptoms. No sooner, however, in such cases, has the amount of compensation been fixed than recovery begins. The medical expert must therefore always be on the alert, and by close inquiry and careful examination he will rarely fail to arrive at a just decision. It is not always so easy to deal with the real malingerer. In many instances he is well acquainted with all the usual tests of vision, and is an adept in the art of deception. When the pretended blindness is said to exist in only one eye the diaphragm test previously referred to is of great value in exposing the fraud. The certainty of the diagnosis depends, however, upon the degree of blindness which is simulated, and when a malingerer pretends to be blind in both eyes, or possesses only one eye, tests are not of much assistance, and the deceit can be found out only by keeping a careful watch over the pretender so as to catch him when he is off his guard.

To those who wish to pursue this subject further I recommend:—

Jameson Evan's admirable review of the most important papers recently written on Compensation after Eye Injuries in the *Ophthalmoscope* for June, 1909.

Magnus and Würdemann, "Visual Economics." Porter, Milwaukee, 1902.

Ramsay, "Eye Injuries and Their Treatment," Maclehose, 1907.

Barnett, "Accidental Injuries to Workmen," Rebman, 1909.

Lawes, "Compensation for Industrial Diseases," Stevens and Sons, Ltd., 1909.

Sym, *Ophthalmic Review*, October, 1906.

Fergus, *British Medical Journal*, December 29, 1906.

Dehenne and Bailliart, *Recueil d'Ophthalmologie*, June, 1908.

Ramsay and Sutherland, *Ophthalmic Review*, 1906, "Spindle-shaped Enlargement of the Blind Spot."

Report of Discussion on the Compensation Act, 1906, British Medical Association Meeting, Belfast, 1909.

